# Characterization of the Wheat Leaf Metabolome during Grain Filling and under Varied N-Supply

**DOI:** 10.3389/fpls.2017.02048

**Published:** 2017-11-29

**Authors:** Elmien Heyneke, Mutsumi Watanabe, Alexander Erban, Guangyou Duan, Peter Buchner, Dirk Walther, Joachim Kopka, Malcolm J. Hawkesford, Rainer Hoefgen

**Affiliations:** ^1^Department Willmitzer, Max Planck Institute of Molecular Plant Physiology, Potsdam, Germany; ^2^European Molecular Biology Laboratory, European Bioinformatics Institute, Heidelberg, Germany; ^3^Plant Sciences, Rothamsted Research, Harpenden, United Kingdom

**Keywords:** wheat leaf metabolism, nitrogen supply, post-anthesis, development, yield

## Abstract

Progress in improving crop growth is an absolute goal despite the influence multifactorial components have on crop yield and quality. An Avalon × Cadenza doubled-haploid wheat mapping population was used to study the leaf metabolome of field grown wheat at weekly intervals during the time in which the canopy contributes to grain filling, i.e., from anthesis to 5 weeks post-anthesis. Wheat was grown under four different nitrogen supplies reaching from residual soil N to a luxury over-fertilization (0, 100, 200, and 350 kg N ha^−1^). Four lines from a segregating doubled haploid population derived of a cross of the wheat elite cvs. Avalon and Cadenza were chosen as they showed pairwise differences in either N utilization efficiency (NUtE) or senescence timing. 108 annotated metabolites of primary metabolism and ions were determined. The analysis did not provide genotype specific markers because of a remarkable stability of the metabolome between lines. We speculate that the reason for failing to identify genotypic markers might be due to insufficient genetic diversity of the wheat parents and/or the known tendency of plants to keep metabolome homeostasis even under adverse conditions through multiple adaptations and rescue mechanism. The data, however, provided a consistent catalogue of metabolites and their respective responses to environmental and developmental factors and may bode well for future systems biology approaches, and support plant breeding and crop improvement.

## Introduction

Among the abiotic parameters necessary for plant growth and development a balanced nutrient supply is essential (Marschner, 1995; Grusak and Dellapenna, 1999; White and Brown, 2010) and insufficient nutrient supply has multiple effects on plant physiology including negatively effecting crop performance and yield (Marschner, 1995). For crop systems, including wheat, nitrogen supply is paramount for optimal yields (Barraclough et al., 2010; Hawkesford, 2014). The use of systems biology for enhancing crop abiotic stress tolerance should be helpful to either improve plant performance at given nutrient supplies by fertilizers or to breed or engineer plants to improve crop ability to deal with insufficient nutrient availability. Our knowledge on detailed molecular processes involved in such abiotic nutrient physiology is incomplete and all additional system wide information will aid in eventual improvement.

The main driver for crop improvement over the last century has been yield. The development of molecular tools supporting plant breeding (Fernie and Schauer, 2009), improved agronomical practices, and the use of chemical fertilisers are the main drivers for yield increases (Hirel et al., 2007, 2011) and mineral nutrient availability is a key factor for obtaining optimal yield (Whitcomb et al., 2014). Cereals have a long history of domestication and wheat, in particular, shows a reduction in genetic diversity (Reif et al., 2005). Elite wheat varieties have classically been bred on high levels of N-fertiliser resulting in a reduced diversity and variation in N use efficiency and N remobilisation efficiency across related populations (Shewry, 2009). However, additional yield enhancements by even further increased nitrogen supply above 200 kg ha^−1^ are limited (Hawkesford, 2014). Increased production demands together with environmental and cost considerations place plant breeders under pressure to develop high yielding crop varieties with improved N use efficiency. An improved understanding of the underlying metabolic processes that relate to nitrogen availability is required to deliver new crop varieties that can make better use of available nitrogen. This applies to N uptake as well as N-mobilization processes during crop development.

Remobilisation of N becomes the dominating pathway during grain filling (Foyer et al., 2011), and contributes to the overall efficient use of nitrogen, though, the accompanying senescence limits further carbon fixation which in contrary would be increased through prolonged photosynthesis due to delayed senescence. In wheat, only a minor portion of grain N is derived from post-anthesis N uptake, while between 50 and 90% of N found in wheat grains is mobilized from the leaf-N or root stored N and the onset of senescence is identified as the main stimulus (Kichey et al., 2007; Gregersen et al., 2008, 2013; Masclaux-Daubresse et al., 2008; Distelfeld et al., 2014). N utilization efficiency (NUtE) for wheat can be defined as grain yield per unit N taken up. In consequence, NUtE improvement is therefore dependent on a coordinated control of the duration of photosynthesis and efficient mobilisation of N upon senescence.

Crop performance with respect to yield and NUtE is primarily determined by breeders through gross agronomical parameters such as grain yield, and N in grain and straw. Here the four lines of a mapping population derived from a cross of elite wheat cultivars which showed variation with respect to senescence and NUtE, were compared. This Avalon × Cadenza doubled-haploid mapping population was assessed in field grown trials at four N levels from zero additional to luxury N supply. Samples were taken in weakly intervals during grain filling from anthesis until final harvest. Agronomical parameters were monitored and compared to the leaf metabolome at sampling time points. The intention of the study was to investigate whether the gross agronomical differences manifest in the metabolome over development and/or in response to N supply. Firstly, this study provides a consistent, and in its density, an unprecedented compilation of wheat leaf metabolome data during grain filling under diverse N-supply. The work demonstrates that N-supply and development are characterised by distinct metabolome patterns. Secondly, the metabolome data was queried to determine whether it would provide metabolites indicative for NUtE which then would bear a potential to be used as markers for breeding as discussed previously (Fernie and Schauer, 2009). The second goal, however, was not achieved due to insignificant differences between the lines other than those being developmentally or environmentally driven. In summary, in this study homoeostasis is affected by development and nutrient availability, but not by genotype, which will be discussed.

## Materials and methods

### Experimental conditions and plant material

Among in total 25 wheat varieties four selected Avalon × Cadenza doubled-haploid lines were grown in a field trial at Rothamsted Research (grown in the Defra WGIN project (www.wgin.org.uk), Harpenden, U.K.) in 2010/11. The population of doubled-haploid individuals was developed by Clare Ellerbrook, Liz Sayers and the late Tony Worland (John Innes Centre), as part of a Department for Environment, Food and Rural Affairs (Defra)-funded project led by ADAS (Provider of agricultural and environmental consultancy, rural development services and policy advice, www.adas.uk). The four lines, 100, 116, 122, and 181 were selected and chosen for this experiment based on their pairwise contrasting responses to nitrogen (line 100-low NUtE, line 116-high NUtE) and post-anthesis senescence (line 181 and 122–early vs. late senescence, respectively) in previous field trials (unpublished). For this experiment, wheat was grown at four N fertilizer levels of 0, 100, 200, and 350 kg N ha^−1^ supplied as ammonium nitrate in two applications and in three randomized replicate block/split-plot design at the Black Horse and Bylands fields (latitude 52°N, longitude 1°W) at Rothamsted Research. The plot size was 15 × 3 m non-destructive for yield measurement and 1.5 × 3 m destructive for sampling. Under UK conditions, N0 (plus 30 kg N/ha of N-min measured in the soil profile in February and any soil N mineralised during the season) would be considered deficient, and N200 sufficient for average yields (8–10 t/ha). Plant growth was monitored for the onset of anthesis. For metabolome and chlorophyll analyses, 10 2nd leaf (from top) samples per replicate were sampled immediately in liquid nitrogen at anthesis and consecutively on a weekly basis thereafter until 5 weeks post-anthesis with nearly complete senescence. Grain yield, straw yield and %N in the grain and the straw were recorded at final harvest at the end of the growing season.

### Determination of leaf greenness

The concentration of chlorophyll (Chl) content per unit area was estimated in attached leaves by taking SPAD-502 (Konica Minolta, Ramsey, NJ) meter readings weekly on the second fully expanded leaf, midway along the leaf blade and half way between the central vein and the leaf edge. Measurements were made on leaves which were subsequently harvested for the metabolite analyses. Leaf greenness is an accepted proxy for leaf nitrogen contents (Lawlor, 2002; Foyer et al., 2003).

### Determination of Chl contents

In order to verify the SPAD measurements which measure spots within the leaf blade, the average Chl content per leaf sample as Chl content of total harvested leaves by Chl extraction, was determined. Whole leaf samples were ground to a fine powder under liquid nitrogen and freeze dried. Chl was extracted twice with 80% and once with 50% ethanol (in water). Extracts were combined. The Chl contents of the combined extracts were measured with a Gemini XPS Fluorescence Micro plate Reader (Molecular Devices, UK). The contents of chlorophyll a (Chl a) and chlorophyll b (Chl b) were calculated with the following formulas: Chl a = 5.21A_665_ – 2.07A_645_, Chl b = 9.29A_645_ – 2.74_A665_, where A_645_ and A_665_ were absorbances at 645 nm and 665 nm, respectively (Arnon, 1949).

### Metabolite extraction

The extraction of metabolites was performed by methanol/chloroform extraction as described previously (Erban et al., 2007). Metabolites were extracted from 30 mg freeze-dried plant material with 360 μl 80% methanol containing 30 μl nonadecanoic acid methylester (2 mg/ml stock in chloroform) for quantitative internal standardisation of the lipid phase and 30 μl of a pre-mixture of sorbitol (0.2 mg/ml methanol) and D-(-)-isoascorbic acid (0.5 mg/ml in H_2_O) used for quantitative internal standardisation of the polar phase. The extract was heated for 15 min at 70°C before 200 μl chloroform was added to the polar phase. 400 μl H_2_O was added to the mixture after shake incubation at 37°C for 5 more minutes, vortexed vigorously before centrifugation to separate the polar and non-polar phases. Aliquots of 100 μl each were dried in a speed-vac. Primary metabolites, amino acid and ion contents were measured from dried extracts.

### Determination of ion contents

Ion extraction from leaf samples was performed as previously described (Watanabe et al., 2013). Dried extracts were dissolved in UPLC/MS grade water and analysed by high-performance anion exchange chromatography with conductivity detection using a Dionex ICS-3000 system (Watanabe et al., 2013). Elution of the ions was performed using a KOH step gradient (flow rate 0.25 ml/min); 0 min, 6 mM KOH; 10 min, 45 mM KOH; 12 min, 55 mM KOH; 17 min, 6 mM KOH).

### Determination of amino acid contents

Dried extracts were dissolved in 70 μl 5 mM sodium phosphate buffer (Na_2_HPO_4_ and NaH_2_PO_4_) pH 6.2. The samples were centrifuged at 20,000 g for 30 min at 4°C. Amino acids were measured by precolumn derivatisation with ortho-phthaldialdehyde in combination with fluorescence detection (excitation wavelength 330 nm; emission wavelength 450 nm) (Lindroth and Mopper, 1979; Rajendra, 1987; Watanabe et al., 2013). Elution was achieved on a Hyperclone C18 ODS column (Phenomenex) connected to a HPLC system (Dionex), applying a solvent gradient with increasing hydrophobicity (buffer A: 0.2% [v/v] tetrahydrofolate, 8.5 mM sodium phosphate buffer, pH 6.8; buffer B: 32.5% [v/v] methanol, 20.5% [v/v] acetonitrile, and 18.5 mM sodium phosphate buffer, pH 6.8; flow: 1.0 mL/min; 0–3 min: 100% A, 5 min: 93% A, 7% B; 14 min: 60% A, 40% B; 18 min: 55% A, 45% B; 28–31 min 100% B, 31.3–34 min: 100% A).

### Profiling of primary metabolism

Profiling of the relative changes of primary metabolite pools by gas chromatography-time of flight-mass spectrometry (GC-TOF-MS) was performed as described previously (Lisec et al., 2006; Watanabe et al., 2013). Multiparallel chromatography data processing and compound identification were performed by TagFinder software (Luedemann et al., 2008) using reference spectra from the Golm Metabolome Database for compound identification (Kopka et al., 2005).

### Statistical analysis

The R statistical language (http://www.r-project.org/) was used for most of the statistical analyses in this study. In the metabolite correlation analysis, each metabolite was normalized by dividing its average from all conditions (the combinations of genotype, treatment and time point). The R “stats” package was used to implement the Pearson correlation analysis. For the principal component analysis (PCA), the biological replicates of each metabolite were firstly averaged as its metabolite expression level, and then each metabolite was normalized by dividing its average from all conditions. PCA was implemented using R package “pcaMethods” (Stacklies et al., 2007). Additionally, the structure of the metabolic network was assessed by examining correlative matrices of metabolites and yield parameters for each genotype to determine which metabolites contribute to improved yield or %N in the grain or straw. The influence of different factors (genotype, treatment, and sampling time point) in the experiment upon individual metabolites was checked using ANOVA with FDR correction (Supplemental Table S1; Benjamini and Hochberg, 1995).

## Results

### Nitrogen supply affected yield attributes and chlorophyll contents in wheat leaves

**Figure 1:**
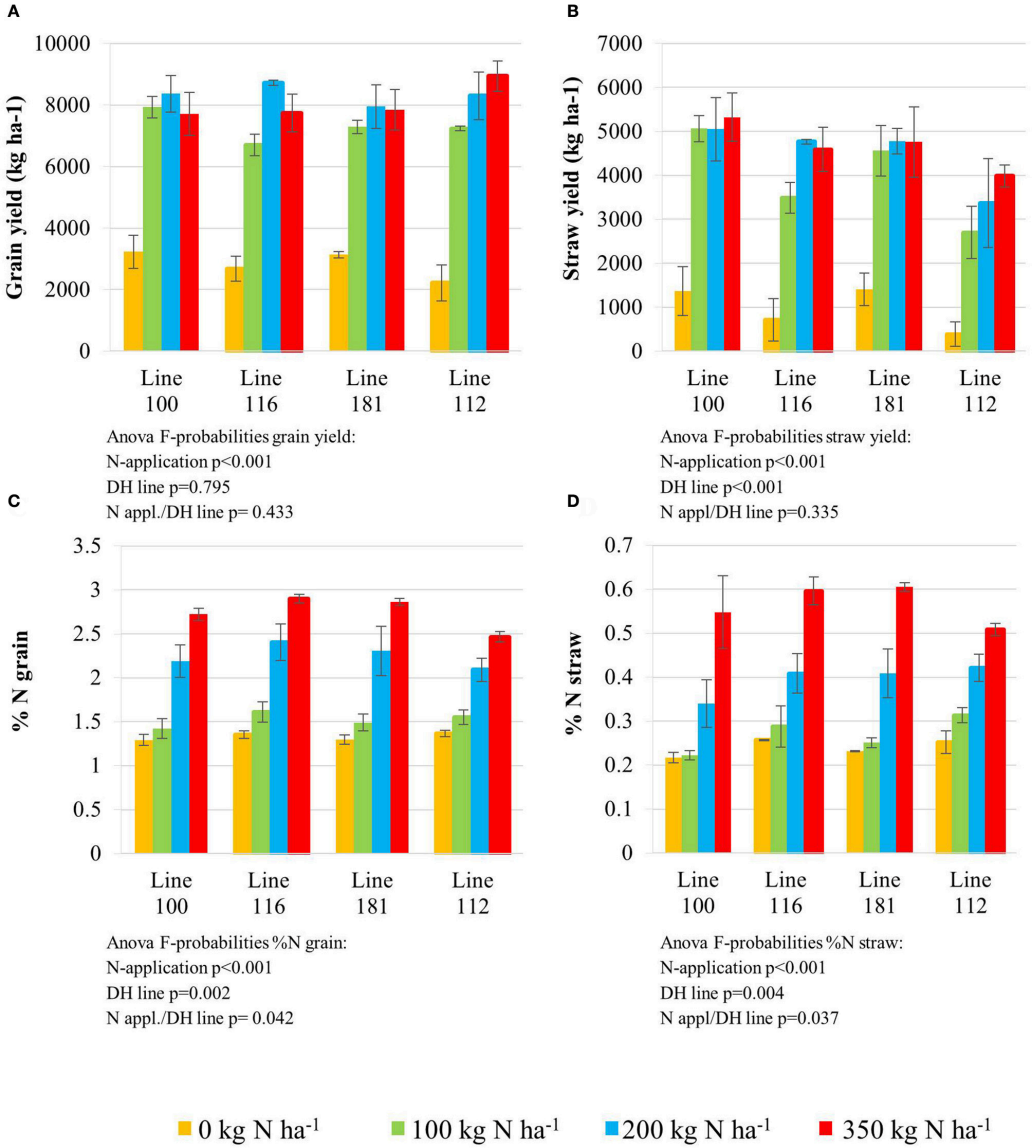
Grain yield **(A)** and straw yield **(B)** determined with the final harvest at the end of the season. Nitrogen concentration (%); LSD = 0.263 measured in the grain **(C)** and straw **(D)**. Doubled haploid lines: line 100 (high NUtE) and line 116 (low NUtE), line 181 (early senescence) and line 112 (late senescence). The values are means (±SE) of three biological replicates. Means followed by different letters among treatments were significantly different according to ANOVA (*P* > 0.05) in Supplemental Table S1.

All plant lines delivered low grain yield (Figure 1A) when grown in plots where zero nitrogen (N0) was administered. An N supply of 100 kg N ha^−1^ (N100) and higher, significantly increased grain yield in all four lines. N application levels higher than 100 kg N ha^−1^ (N200 and N350) did not further improve grain yield significantly for lines 100 and 181, while lines 116 (higher NUtE) and 112 (delayed senescence) achieved slight but significantly higher grain yields at N200 and N350. It is noteworthy that no yield increase could be achieved with applications beyond 200 kg N ha^−1^ as an application of 350 kg N ha^−1^ did not further affect performance under field conditions. Although no increase of grain yield in kg ha^−1^, the high N supply increased nitrogen contents in the grain (Figure 1C) and in the straw (Figure 1D) indicating that additional N is taken up even beyond 200 kg N ha^−1^ and contributes to the yield components but not to higher yields in total probably due to sink limitations for carbohydrates.

The various yield parameters have been determined post-harvest. The working hypothesis of this study was to investigate and identify the leaf metabolite composition of four contrasting wheat doubled haploid lines during seed filling under four different nitrogen application regimes to possibly identify potential predictive markers related to NUtE. Further, this analyses would in parallel yield a catalogue of metabolites responsive to (i) different levels of N-supply, (ii) developmental series during grain filling, and (iii) reveal genotype specific differences i.e., markers.

**Figure 2:**
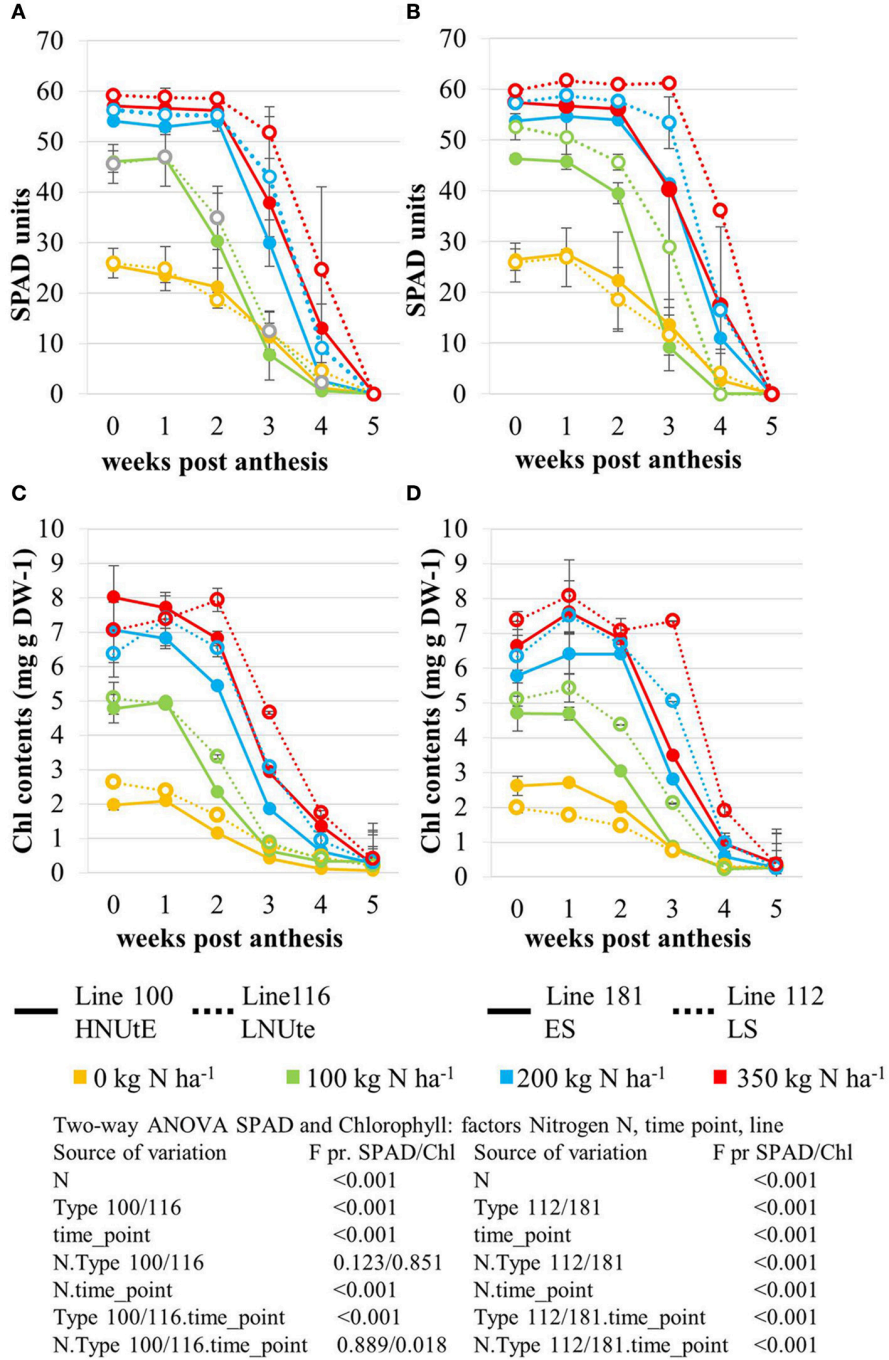
Leaf Chlorophyll content expressed as relative SPAD values measured in the NUtE inbred lines **(A)** and the senescence inbred lines **(B)**. Changes in total chlorophyll (μg/mg DW) content in the second leaf following anthesis. Data represent mean values of three biological replicates for each time point measured in the NUtE inbred lines **(C)** and the senescence inbred lines **(D)**. Doubled haploid lines: line 100 (high NUtE) and line 116 (low NUtE), line 181 (early senescence) and line 112 (late senescence). The values are means (±SE) of three biological replicates. Means followed by different letters among treatments were significantly different according to ANOVA (*P* > 0.05) in Supplemental Table S1.

The nitrogen status of the wheat canopy under agricultural conditions may be assessed by greenness of the leaf, which serves as proxy for N content (Lawlor, 2002; Foyer et al., 2003). Leaf greenness is determined by chlorophyll content. Therefore, leaf chlorophyll content of the sampled leaves was determined by SPAD measurements *in vivo* on site (Figures 2A,B) and by chlorophyll extraction of the sampled leaf material (Figures 2C,D). SPAD and total leaf chlorophyll measurements yielded matching results despite the fact that SPAD measures a focal area on the leaf while chlorophyll extraction averages chlorophyll content of the whole leaf sample. The N application rates had a strong effect on the total chlorophyll contents in the leaves showing a positive N dose-response relationship in all lines and the concentrations were quite comparable between all lines at anthesis being directly correlated to the amount of N supplied (Figures 2C,D). For all lines the reduction of chlorophyll content during seed filling is obvious reducing leaf chlorophyll to very low levels 5 weeks post-anthesis no matter how high the levels were previously in response to N supply at anthesis. The ANOVA analysis indicated significant differences all over between the two lines (100, 116) seen at high N supplies (N200, N350) with high NUtE line 116 displaying higher leaf chlorophyll levels suggesting higher N contents than the contrasting line 100 (Figures 2A,C) as confirmed as higher NUtE from previous field experiments (unpublished). No or low significance in chlorophyll content was seen for the interaction of both lines with N-application and time (Figure 2). This was different between the late senescing line 112 showing at N100, N200, and N350 slightly higher chlorophyll levels than line 181 as well as a notably shift of chlorophyll contents to later time points. The statistical significant interaction between the lines and N-application including time-point of harvest corroborate the field observations on line 112 as being a late senescing line compared to 181. This would provide an extended time for photosynthesis respectively higher photosynthetic rates at a given time point for line 112 vs. line 181 (Figures 2B,D). This, however, leads only to slightly higher grain yield (Figure 1A). Thus, despite detectable differences in chlorophyll content or senescence timing, the effects on grain yield are minor between the four lines. Low N (N0) affects grain yield massively in all lines, while already N100 allows to almost reach the maximum yield level for grain and straw (Figures 1A,B). For the low NUtE line 116 the reduced yields at N100 could not be verified as statistically significant. With respect to grain yield, there were significant differences in straw yield between the lines but the response ao all lines towards N-application were comparable (Figures 1A,B). Despite overall high yield (grain and straw (Figures 1A,B) percentage of N in grain (Figure 1C) or straw (Figure 1D) is as low in N100 as N0 and is generally directly dependent and responsive to applied N levels with some significant differences between the lines in relation to the to N-application.

### N-supply and plant development interact in re-programing primary metabolism

To obtain further insights into metabolite pools that affect yield under low-N, the metabolite composition were scrutinised for indicative differences among metabolites of all four lines under the four N conditions and over developmental time. A metabolite analysis was employed yielding in total 108 annotated metabolites to dissect wheat leaf metabolic states under the four N application rates over the post-anthesis developmental period of 5 weeks. The metabolite data were subdued to a principal component analysis (PCA).

**Figure 3:**
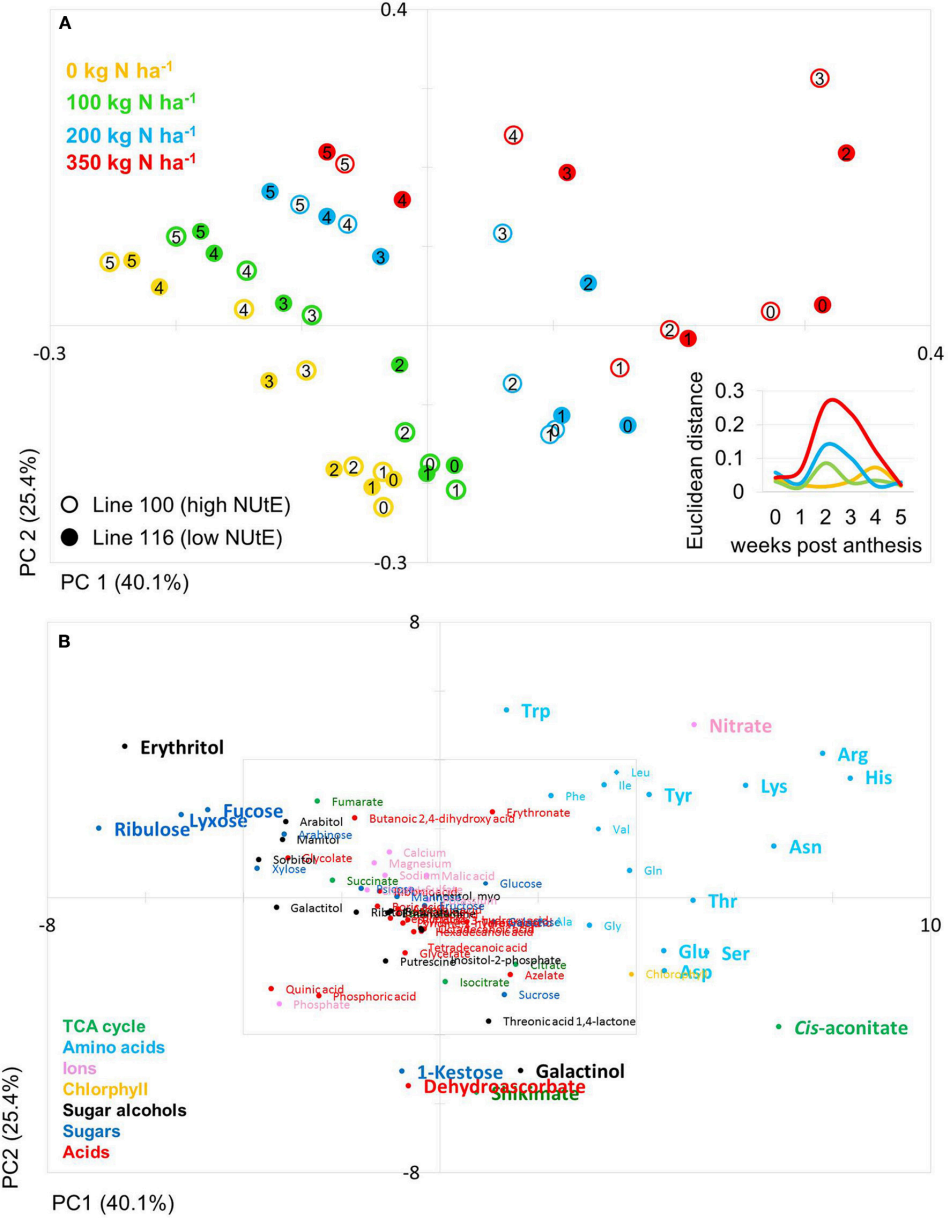
Principal component analysis (PCA) of metabolite data. **(A)** PCA score plot of primary metabolite profiles differentiating the high and low NUtE lines. Circles indicate averages of biological replicates of wheat leaf material harvested at 0, 1, 2, 3, 4, and 5 weeks past anthesis, respectively. Open symbols represents line 100 (high NUtE) and solid symbols represents line 116 (low NUtE). The insert depicts the Euclidian distances between the PCA loadings of line 100 and line 116 at consecutive time points. **(B)** PCA loadings plot of all measured primary metabolite profiles for DH lines line 100 (high NUtE) and line 116 (low NUtE). The algorithm was applied to 108 annotated primary metabolites detected in leaf material. PC, principal component; NUtE, nutrient utilisation efficiency.

The PCA score plot of the comparison of line 100 and 116 (high and low NUtE, respectively) showed that principal component 1 (PC1; 40.1% of variance) separates data mainly with respect to N levels while PC2 (25.4% of variance) separates data to due time point of harvest and thus developmental stages, essentially plant age (Figure 3A). It is notable that while at N0, N100 and N200 data pairs of the two lines are quite related as being positioned close to one another indicating low variations within the dataset and hence, between the lines (genotypes) at the level of metabolites. At N350 with a luxurious N supply of 350 kg N ha^−1^ the components tend to be spread quite far from one another without any clear tendency for any of the two lines which indicates a high variation within the data set. The calculation of the Euclidian distance of the data set in relation to N supply and time point (Figure 3A insert) also displays that at N350 the highest variance can be observed which is graded down via N200–N100. The highest variance can be observed between 2 and 3 weeks post-anthesis. At this time also the chlorophyll data show the biggest differences between lines (Figures 2A,C).

**Figure 4:**
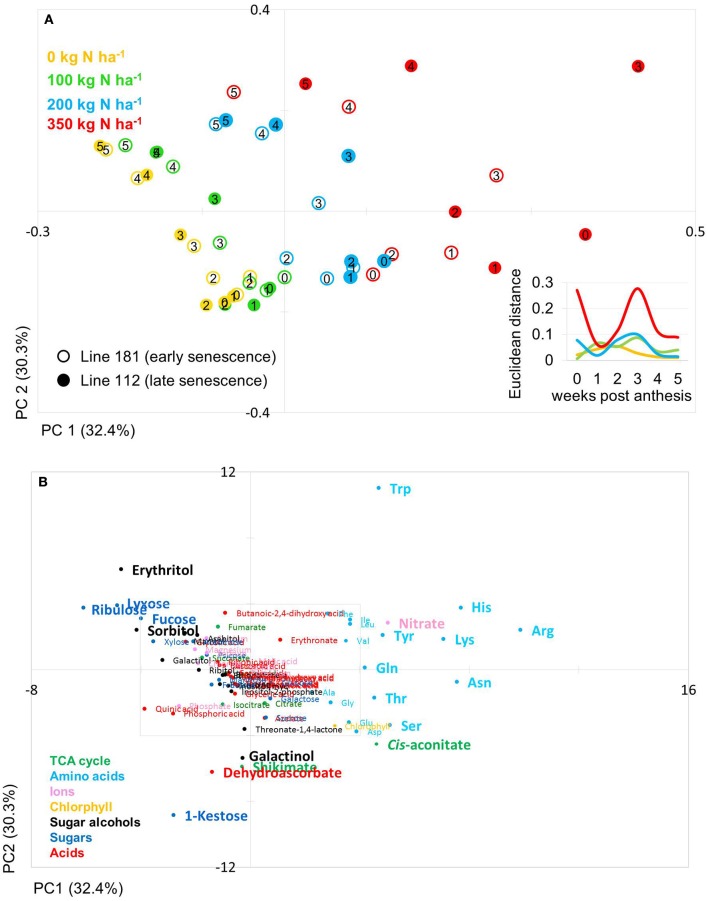
Principal component analysis (PCA) of metabolite data. **(A)** PCA score plot of primary metabolite profiles differentiating timing of senescence. Circles indicate averages of biological replicates of wheat leaf material harvested at 0, 1, 2, 3, 4, and 5 weeks past anthesis, respectively. Open symbols represents line 112 (late senescence) and solid symbols represents line 181 (early senescence). The Insert depicts the Euclidian distance between the PCA loadings of line 112 and line 181 at consecutive time points. **(B)** PCA loadings plot of all measured primary metabolite profiles for DH lines line 112 and line 181. The algorithm was applied to 108 annotated primary metabolites detected in leaf material. PC, principal component.

The PCA score plot of the comparison of line 181 and 112 (early and late senescence, respectively) shows that principal component (PC1; 32.4%) separates data due to N levels while PC2 (30.3%) separates data mainly with respect to time and thus developmental stages (Figure 4A). Also for these lines data pairs at N0, N100, and N200 are being closely positioned to one another indicating low variations within the dataset and hence, between the lines at the level of metabolites. At N350 with a luxurious N supply of 350 kg N ha^−1^ the components tend to be spread quite far from one another. Here, the slow senescing line 112 is clearly much stronger separated by PC2 (N-supply) from its corresponding data point of line 181. This indeed indicates a correlation between delayed senescence and effect of N application on line 112. The calculation of the Euclidian distances of the data set in relation to N supply and time point (Figure 4A insert) displays that at N350 the highest variance can be observed while all other N supplies show little variance between the lines for N0, N100, and N200. Interestingly, the highest Euclidian distance is calculated for time point zero, the onset of anthesis and for 3–4 weeks after anthesis, visible, though low, also for N100 and N200. This later peak of variation between the lines 116 and 181 and also in comparison to 100 and 116 can again be related to the later senescing phenotype which leads to an about 1 week shift in metabolic signature differences as displayed already by the chlorophyll measurements (Figures 2B,D).

The PCs are determined by the contribution of all metabolites. The impact of a distinct metabolite on the principal component is captured in its loading value. The plots of the PCA loadings (Figures 3B, 4B) display the contribution of single metabolites to the global separation seen between samples (Figures 3A, 4A). It becomes obvious from these plots that the differences are mainly driven by a small amount of metabolites. Interestingly, the shared metabolites with the highest loading plot scores are almost identical between the four lines being the amino acids Trp, His, Arg, Lys, Asn, Tyr, Ser, the sugar alcohols erythritol and galactinol, the sugars lyxose, ribulose, fucose and the TCA intermediate cis-aconitate are also influencing PC1 in all four lines. Nitrate is as well a clear determinant, as the environmental component is driven by N availability; however, the proxy for nitrate, chlorophyll, is just below the selected threshold. As such, chlorophyll might be interpreted rather as how efficiently N is used for building the photosynthetic apparatus, i.e., as a proxy for N use, but not necessarily as a proxy for leaf nitrate accumulation because other factors like plant growth stage, cultivars, soil water and deficiency of nutrients other than N can affect chlorophyll content. Glu, Gln, Asp, and Sorbitol vary between both contrasting pairs, and being just immediately below or above threshold (4 eigenvalues). PC2, representing the developmental separation of the data is characterised by 1-kestose, dehydroascorbate, galactinol, and shikimate. Also the amino acid Trp shows a strong developmental component. Again the metabolite loadings overlap between all four lines.

**Figure 5:**
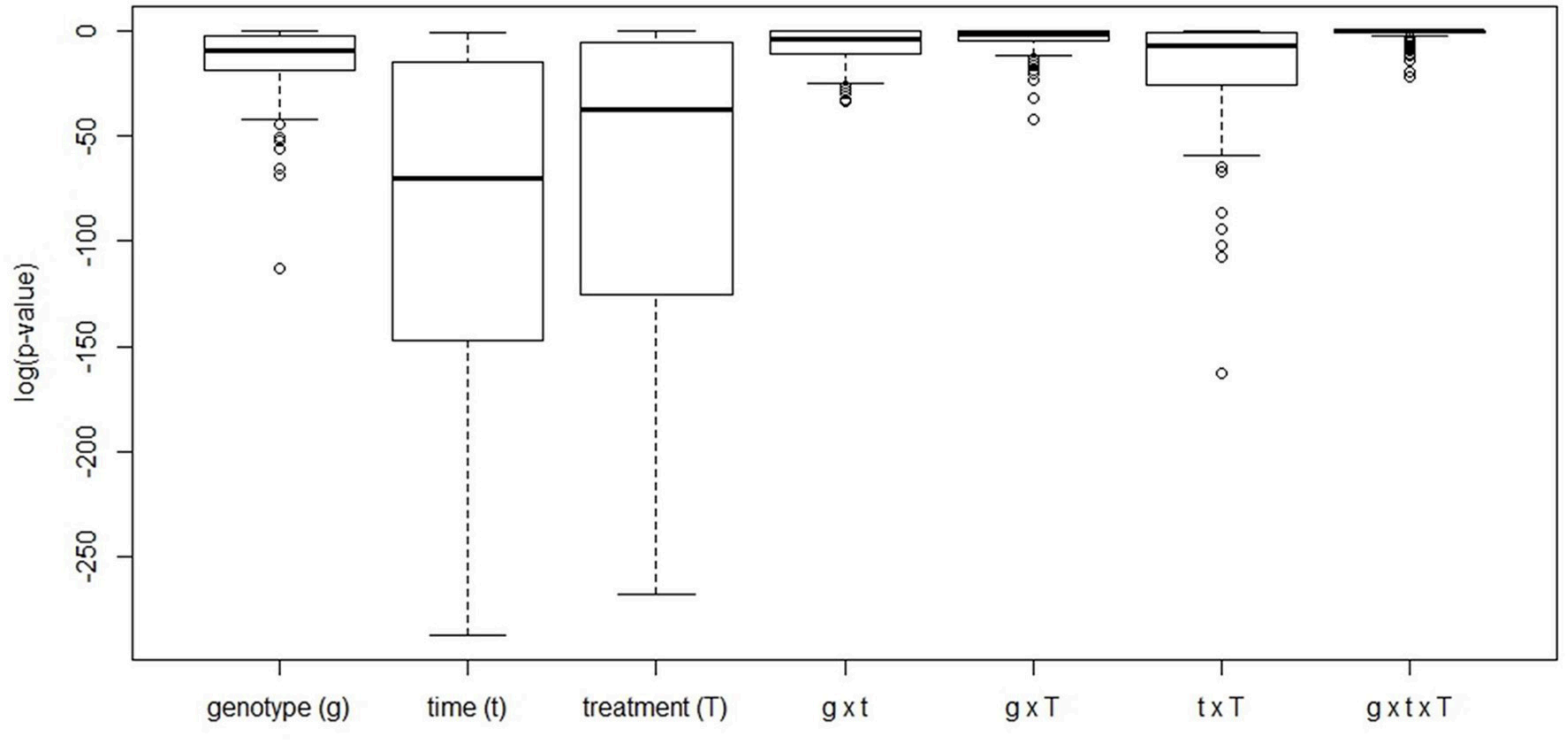
Boxplot of the FDR-corrected *p*-values obtained from a three-way ANOVA analysis performed for every metabolite available for analysis. For every metabolite, levels were scaled by dividing by its average value across all conditions with subsequent log-transformation to render data normally distributed.

**Figure 6:**
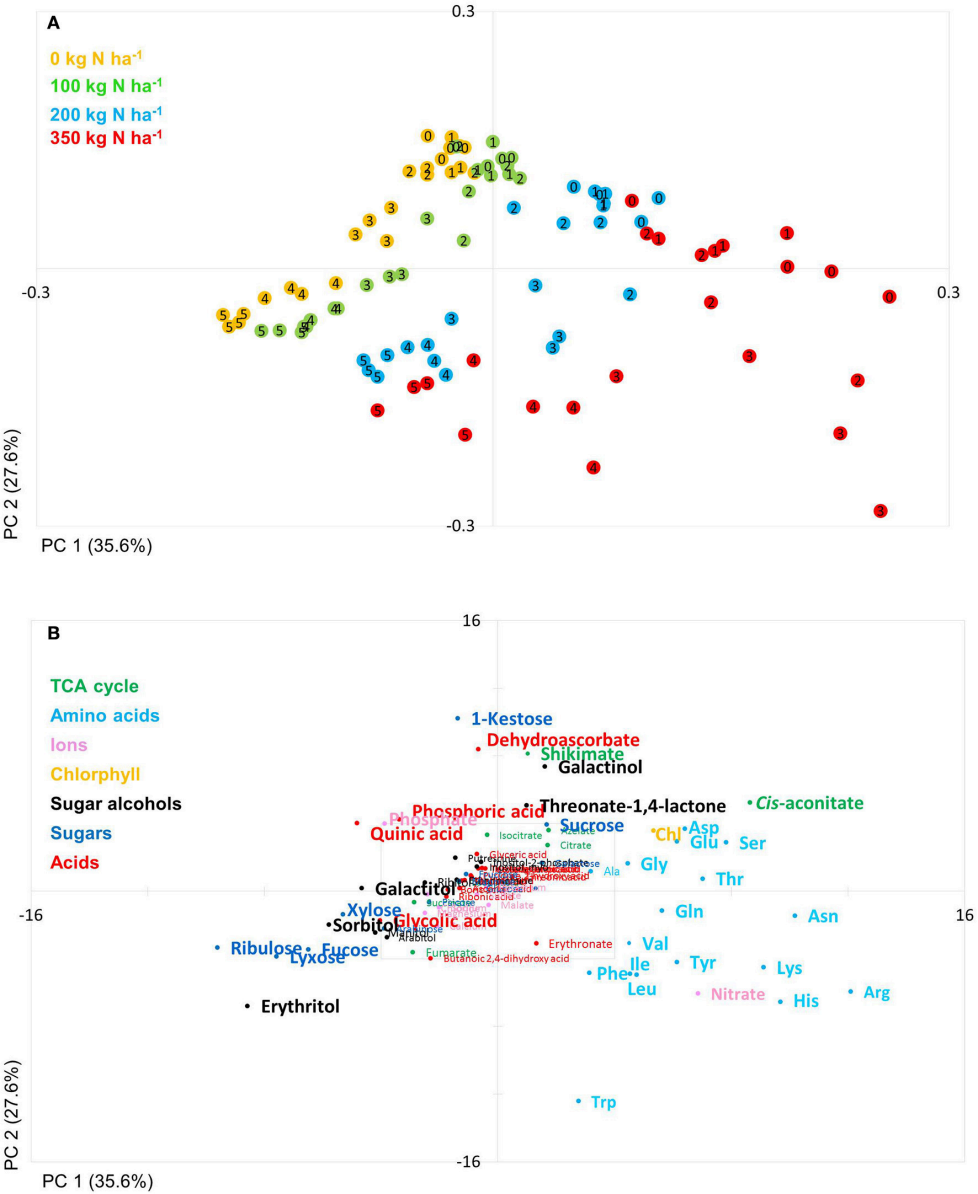
Principal component analysis (PCA). PCA score plot **(A)** and PCA loadings plot **(B)** of all measured primary metabolite profiles. The algorithm was applied to 108 annotated primary metabolites detected in leaf material. PC, Principal component; weeks post-anthesis are numbered: 0, 1, 2, 3, 4, and 5.

Based on a three-way ANOVA analysis including as factors (i) genotype, (ii) time (time pont of harvest, development), and (iii) treatment (N-supply environment), the factors time and treatment were identified as influencing metabolite levels most significantly. By comparison, genotype had a negligible effect (Figure 5) and as a consequence, only marginal genotypic differences between the four lines were revealed at the metabolome level (Figures 5, 6). Therefore, the data are not justifiably suited to derive markers for breeding purposes, at least among the 108 annotated metabolites of this study which comprise metabolites of primary wheat metabolism and nutrient ions. The marginal disparity is probably based on the fact that both parent lines were elite lines (Reif et al., 2005; Lopes et al., 2015). Conceding that the genetic component of the response to varied nutrient supply was small relative to the environmental component, we decided to pool the complete data set and rather treat the joint data set as a joint wheat response panel towards nitrogen supply in relation to seed filling time. The pooled data display expectedly a similar pattern of the agronomical parameters (Supplemental Figure S1) and of the leaf sample chlorophyll contents (Supplemental Figure S2) as the individual displays (Figures 1, 2) The respective PCA analysis (Figure 6) provides a comprehensive dataset allowing to describe metabolic changes for wheat N supply response and the seed filling period. PC1 (35.6%) describes variance in response to nitrate supply and PC2 (27.6%) is driven by development (Figure 6A). The components are clearly separated indicating distinct metabolic differences between the different states (nitrogen and time). N0 and N100 fall close together conforming that these conditions can be regarded as N-deficient, i.e., a supply below optimal supply which would allow reaching the growth and yielding optimum. Again, at N350 which represents over-fertilisation the PCA of the data set indicated high diversity within the data set. With the fused data more metabolites contribute significantly to the principal components as shown in the PCA loadings plot (Figure 6B). As PC1 describes N response it is notable that 15 amino acids, nitrate and chlorophyll fall into this group. Additionally, cis-aconitate, the sugar alcohols galactinol, sorbitol, erythritol, the sugars xylose, ribulose fucose and lyxose, quinic acid, and phosphate display an N-responsive phenotype. While PC2 (development) is most strongly characterised by the stress related compounds 1-kestose, dehydroascorbate, shikimate, threonate-1,4-lactone, erythritol and galactinol, and additionally sucrose. The amino acid Trp shows a strong developmental response component which previously has been observed for senescence processes during grain filling. (Nishizawa et al., 2008).

### Co-behaviour analysis between N-supply and development

**Figure 7:**
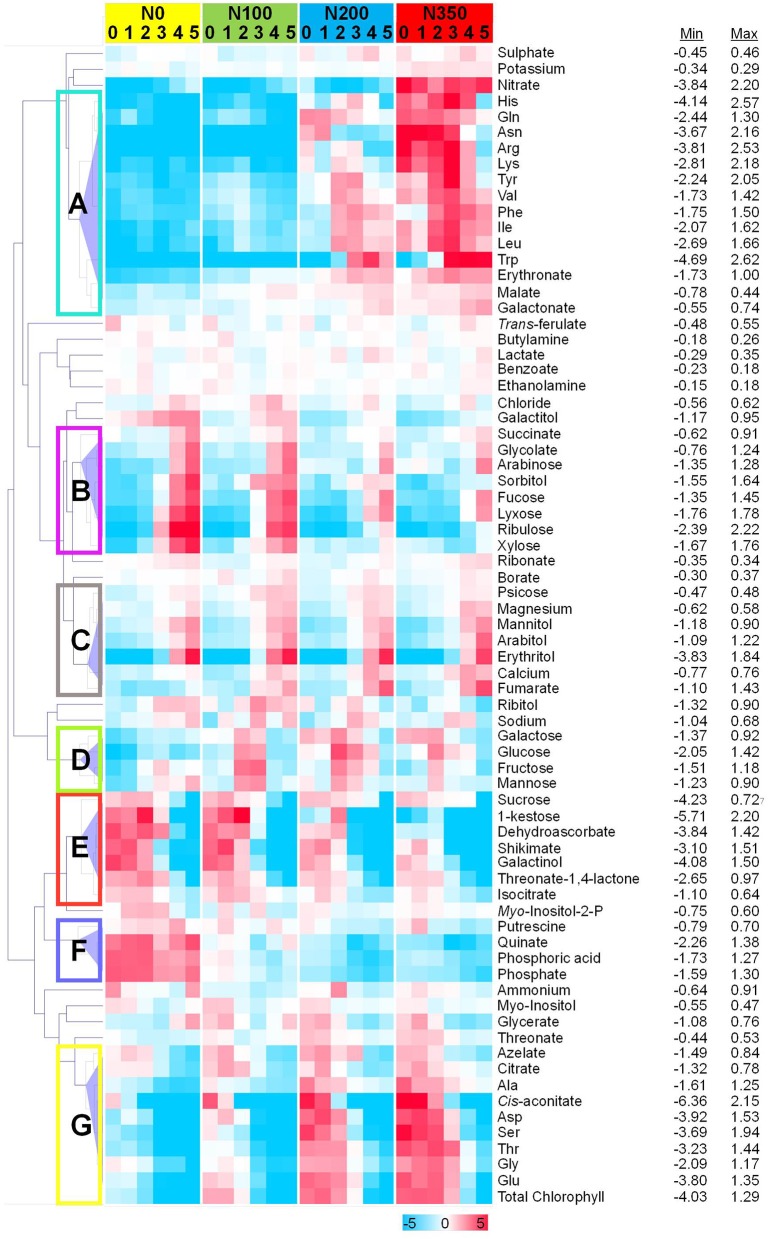
Differentially abundant metabolites in wheat leaves. Hierarchical cluster analysis separated metabolites into different classes subject to their response pattern. Absolute values as recorded were averaged and normalized to the average of each metabolite. Log_2_-fold values are presented by the false color code. Hierarchical clustering analysis separated metabolites into seven distinct clusters: metabolites displaying a strong N dependency, but showing a biphasic response over the developmental period **(A)**; metabolites decreasing with increasing N level, but then showing increased levels over the developmental period **(B)**; those increasing over both increasing N level and developmental period **(C)**; metabolites showing a biphasic pattern over both increasing N level and developmental period **(D)**; those decreasing with increasing N level and developmental period **(E)**; metabolites decreasing over increasing N level, and having a biphasic response over developmental period **(F)**; and those increasing over increasing N level, while decreasing over developmental period **(G)**.

To assess metabolic signatures of distinct metabolites a hierarchical cluster analysis was employed at all 4 N levels and within respective N-levels the six samplings are depicted (Figure 7). At least 7 distinct response clusters (denoted A–G in Figure 7) are separated by the hierarchical clustering algorithm.

For this, the absolute values were averaged and normalised to the average of each metabolite. Log_2_-fold transformed values for each metabolite were clustered by computing the Euclidean distance on average linkage distance which defines an unweighted pair-group method using arrhythmic averages, i.e., the average distance between any two members (D'haeseleer, 2005). The hierarchical dendrogram revealed co-modulated metabolites. The metabolite responses that were largely driven by N level and developmental program could be divided into seven distinct clusters termed A to G (Figure 7). Interestingly clustering is displayed as well as close neighbourhood in the PCA loadings plot (Figure 6B). “Cluster A” comprises a group of amino acids (His, Gln, Asn, Arg, Lys, Tyr, Val, Phe, Ile, Leu, Trp together with erythronate and malate) related in its response behaviour over time and nitrogen supply to nitrate and “Cluster G” being related to chlorophyll consisting of the amino acids Ala, Asp, Ser, Thr, Gly, and Glu together with cis-aconitate and citrate. Also the previously identified stress related compounds (1-kestose, dehydroascorbate, shikimate, galactinol, threonic acid 1,4-lactone with addition of isocitrate and sucrose) cluster together in “Cluster E.”

Some general response patterns can be derived. Clusters A and G comprising mainly amino acids, nitrate and chlorophyll i.e., N-rich substances accumulate at N200 and N350 and decrease over time, probably indicating transport to seeds. Stress related compounds (Cluster E) decrease at all N levels over time and show higher abundances the less N is supplied, i.e., stress compound accumulation occurs under reduced N-supply. Actually, “Cluster F” consisting of putrescine, citrate, and phosphate could be added to this group. Cluster F is strongly induced under N0 only, thus, being markers for nitrogen starvation in wheat leaves. Clusters B and C (mainly sugar alcohols) display metabolites which are senescence related (Bowne et al., 2012) and accumulate at late sampling time points. In cluster B metabolite accumulation is enhanced additionally through reduced N supply while cluster C metabolites are only senescence related.

### N application rates effects on ion homeostasis

The levels of several ions, among them nutrient ions, were also affected by N application rates and by developmental program (Figures 6, 7). Leaf nitrate contents were low at the N0, N100, and N200 application rates and accumulated to high levels only at N350 indicating that up to N200 applied nitrogen was used for biosynthesis while only when supply exceeded needs nitrate was stored in the vacuole (Martinoia et al., 1981). Phosphate displayed an inverse relation to nitrate and accumulated to high levels in leaves of plants grown at N0. Sulphate and potassium contents did hardly respond to nitrate supply or developmental state and only showed a slight accumulation at N350. Chloride and Magnesium did hardly respond to N supply and accumulated to the end of senescence though but slightly. Ammonium showed no obvious correlation with N supply or to the time of sampling.

### Metabolites positively correlating to increased yield

**Figure 8:**
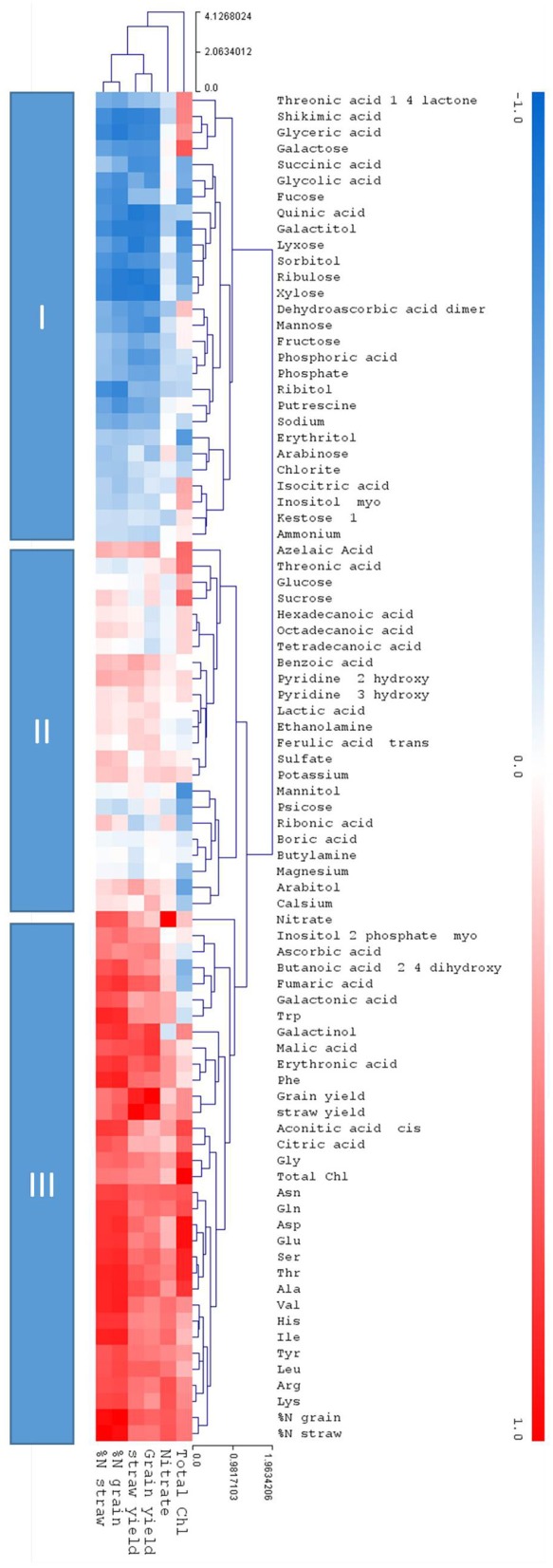
Clustered heatmap depicting correlations between each of the measured metabolites and the core physiological parameters; Total ChI, Nitrate, grain, and straw yield and %N. in the grain and the straw. The metabolites are placed in remote clusters using Pearson Correlation. The assigned color indicates the strength of a particular correlation between two parameters, blue for negative correlations, and red for positive correlations as depicted in the colour key (−1 < 0 < 1). The values were generated in R (http://rstudio.com/) and the heat map was constructed using the MultiExperimentalViewer software (http://www.tm4.org/). Hierarchical cluster analysis separated metabolites into different classes subject to their correlation to the core parameters: Cluster I depicts negative correlations to the core parameters, cluster II show a neutral or inconsistent correlations, and cluster III depicts positive correlations to the core parameters.

While hierarchical clustering (Figure 7) signified the relative relations of distinct metabolites the correlation analysis calculated the degree of relatedness (correlation) using correlation indices over the complete dataset (Figure 8). The correlation to the core parameters of this study, namely chlorophyll content, nitrate, and the agronomical parameters grain yield, straw yield, %N in grain, and %N in straw, identified three response clusters (Figure 8).

Cluster I is negatively correlated to the core parameters, cluster III is positively correlated, while cluster II is mainly positively correlated but metabolites partially show a neutral or inconsistent correlation. In clusters I and III the prevalent correlation was towards nitrate and the agronomical parameters grain yield, straw yield, %N in grain, and %N in straw. Correlation to chlorophyll content was partially deviating from this pattern, again indicating that chlorophyll is a rather a proxy for sufficient N-supply for biochemical synthesis but not for leaf nitrogen levels *per se*. The negatively correlated cluster I contained, among other metabolites, the stress and senescence related metabolites of clusters B, E, and D (Figure 7), while the N and development dependent metabolites of clusters A and G are found in the positively correlated cluster III.

## Discussion

Nutrient availability eventually determines yield, together with other abiotic factors such as water, CO_2_ and light. Any imbalance will cause molecular adaptation processes and will eventually result in decreased crop yield or crop quality (Whitcomb et al., 2014). Crop taken from the field at harvest removes nutrients additional to other losses as leaching or inaccessibility due to binding of e.g., phosphate to soil minerals (Sharpley et al., 2015). Therefore, various agricultural practices have been developed to take account of such depletion with mineral nutrient fertilizer application being the main approach in modern agriculture to obtain optimal crop (Pretty, 2008). In parallel, plant breeding thrives to improve plant performance under the respective agricultural environments (Thro and Zankowski, 2003). Yield increases, as the primary goal of breeding and also necessary in view of the approaching population increase, nevertheless are achieved at incremental steps only. Yield is a multifactorial trait depending on the genetic makeup of the crop and of the environmental conditions, including nutrient ion availability. Next to the highly efficient breeding and selection processes a better molecular understanding of physiological and biochemical processes in crop plants bears a potential to add a knowledge based component to plant breeding. Furthermore, it is necessary to investigate such processes under field conditions, to identify traits or processes relevant under natural multifactorial conditions.

The presented experiment aimed at determining the metabolome of field grown bread wheat using four lines from a segregating elite line cross (parent cvs.: Avalon and Cadenza) and a doubled haploid population derived of this cross previously scored for agronomical parameters. The lines, pairwise varying in NUtE (100, 116) and senescence timing (112, 181), were exposed to four different N fertilization regimes providing no (N0) additional N allowing the plant to only grow on residual remaining soil nitrogen which essentially corresponds to nutrient depletion and successively increased N (kg N ha^−1^) of N100, N200, and N350. N200 is the common amount applied in the UK, while N350 is an oversupply. The second leaf from top was sampled and a metabolome analysis, mainly targeting primary metabolites and ions, was performed which yielded 108 annotated compounds. Non-annotatable peaks were not taken into consideration. The second leaf was chosen as a representative of wheat canopy supply to the grain not ignoring the fact that also other tissues contribute to grain filling (Sanchez-Bragado et al., 2016).

This accounts for a comprehensive catalogue of compounds with their respective contents at the six harvesting time points at weekly intervals between anthesis and full leaf senescence at harvest. The primary goal was to assign specific metabolites indicative for the investigated traits, higher NUtE [line 116 (low) vs. 100 (high)] and delayed senescence [line 181 (early) vs. 112 (late)] which have been determined phenotypically in previous investigations of the separating population from the Avalon × Cadenza cross. Though metabolic signatures could be assigned for either varied N supply (environment) or sampling time point (development) metabolites which were valid enough to define them as metabolic markers describing the trait (genotype) and potentially serving as marker for future plant breeding, could not be identified. There were differences of metabolite contents detectable between lines; however, these were mainly gradual in nature and not decisive enough to provide a basis for a high throughput measuring system necessary for plant breeding.

Metabolite determination has previously been shown to have the potential to provide such markers (Fernie and Schauer, 2009; Lisec et al., 2011). Therefore, it can be assumed that the failure to do so in this experiment was probably due to very low genetic diversity between the parent wheat lines as these were both commercially used elite cultivars. Wheat genetic diversity underwent a substantial constriction during breeding history (Zamir, 2001; Fu, 2015). Additionally, plants tend to retain nitrogen homeostasis as long as possible through various adaptation mechanisms such as increased uptake of N, altered mobilization patterns, reduced growth and to keep metabolite concentrations constant despite abiotic stress conditions as has been shown for example for Arabidopsis under N-depletion (Tschoep et al., 2009). In consequence, this results, as we observed also in this study, in a fairly stable metabolome irrespective of other changes e.g., at the level of transcriptome, enzyme activity, or flux. Disequilibrium eventually arises only when adaptation and rescue mechanisms cannot compensate for the nutrient depletion caused reduction of the respective nutrient dependent metabolites. The nutrient regime (environment) driven metabolome is here overlaid by a developmental component (cf. Figure 6) as nutrients are mobilized in the leaf, converted to transportable metabolites, and transported from the leaf canopy, here second leaf from top, to the developing grain. This is in this study especially well observed for all amino acids (Figure 7 cluster A,G). Though this has been described previously it supports correctness and applicability of the data achieved in this experiment (Simpson and Dalling, 1981; Masclaux-Daubresse et al., 2008; Zhao et al., 2015). Thus, the biggest differences in the metabolites of clusters A, G, and F (Figure 7) occur depending on the N supply with A and G being low and F being high, especially under the N depleted N0 condition, but also, although more moderate, under N100 which both constitute an insufficient N supply as also manifested in reduced yields and %N accumulation in tissues at N0 and N100 (Figure 1; Supplemental Figure S1). At N0 and N100 free amino acid contents are low in the leaf tissues due to the insufficient N supply and assumingly synthesized amino acids being exhaustively incorporated into protein. Further, numerous genes involved in amino acid biosynthesis are strongly repressed in N starved wheat leaves (Howarth et al., 2008). At N200 and N350 free amino acids accumulate (Figures 7, 8) before being reduced in tissues with progressing leaf senescence. At later sampling time points the free amino acid contents are decreased, assumingly being transported to the grains or metabolized for other purposes (Gregersen, 2011).

In consequence the three-factorial analysis of lines (G), nitrogen supply (E), and development (D) could be reduced to the two factors environment (E) and development (D) by fusing the dataset of all determined lines. Even though this dataset could not provide genotypic markers indicative of the traits under study; it provided a quite exhaustive catalogue of primary metabolites and ions which will be helpful in future for understanding wheat physiology in general and under field (natural) conditions in response to nutrient availability.

**Figure 9:**
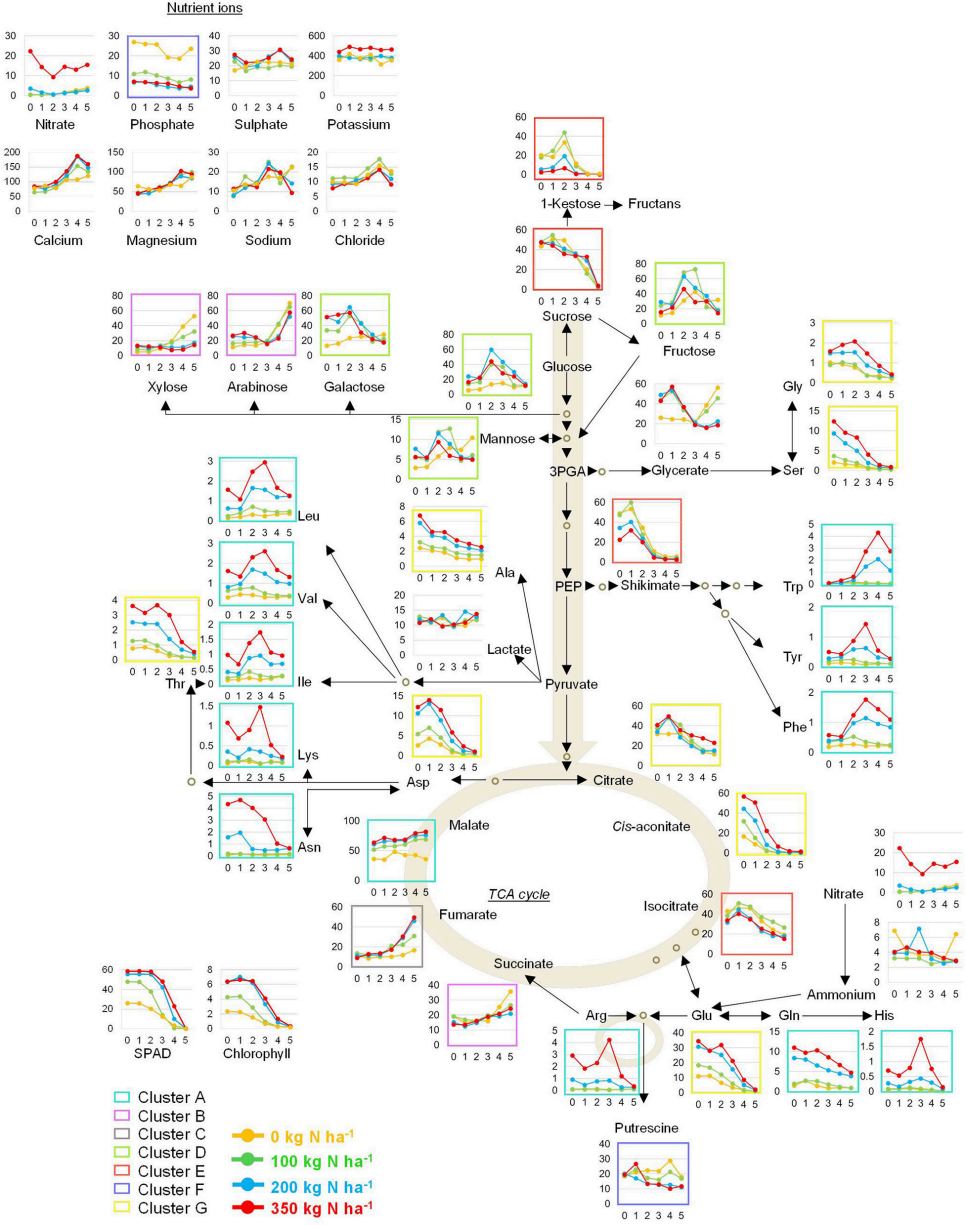
Pathway map of the primary metabolism in wheat. The inserted graphs depict absolute values for amino acids (HPLC) and ions (IC) expressed as). l mol gDW^−1^. All other primary metabolites were determined by GC-MS analysis and are expressed as relative peak height gDW^−1^. The coloured frames correspond to clusters depicted in Figure 4.

Individual metabolite signatures over time and in response to N supply were plotted next to a pathway map depicting the core primary metabolism (Figure 9). The colour code of the clusters shown in Figure 6 was employed here as well. This map together with the individual responses of the four lines (Figures 1–4) provides a consistent catalogue of the highly complex metabolite responses in wheat during grain filling and for varied N application levels.

Most of the metabolites associated with sugar metabolism, such as the monosaccharides fructose, glucose, and galactose displayed a response pattern strongly dependent on the N-treatment increasing strongly up to 2 weeks post-anthesis (wpa). The decreases in glucose and fructose levels occurred from 2 wpa onwards and were more apparent in N fertilised plants. Furthermore, glucose and fructose peaked earlier in plants grown at the high N (N200 and N350) than in those grown at low N (N0 and N100) levels. This response of glucose was absent in the N0 plants suggesting that these plants have impaired photosynthesis rates, as also chlorophyll levels are low, incapable of building up glucose and fructose reserves. However, even under these conditions (N0) sucrose accumulated, though total biomass was impaired and grain yield reduced. Such biomass reduction due to insufficient nutrient availability is a common response (Sieh et al., 2013; Whitcomb et al., 2014). The disaccharide sucrose continuously decreased over the developmental period, independent of the N application rate. By contrast, pentose sugars such as ribulose, lyxose, xylose and arabinose as well as the hexose sugars fucose, and to a lesser extent psicose increased over the developmental period. These sugars negatively correlated to leaf nitrate and yield phenotypic traits.

A tight interconnection exists between carbon and nitrogen metabolism (Stitt et al., 2002; Foyer et al., 2003). Previous reports in Arabidopsis could associate the timing of leaf senescence to glucose and fructose concentrations in the leaves (Tschoep et al., 2009; Watanabe et al., 2013). If N is not available in sufficient amounts, metabolites accumulate to a much lower extent, especially the amino acids for obvious reasons. The carbohydrates sucrose and fructose were not affected at all or only at suboptimal (N0, N100) N-supply levels in case of glucose while the fructane precursor 1-kestose accumulates to higher levels at N0 and N100. This is in agreement with the accumulation of carbohydrates reported for N-starved maize leaves (Schlüter et al., 2012). The induction of sugar levels is particularly important under low-N. If N is not available, N is remobilized from the photosynthetic active tissue reducing their photosynthetic capacity and accelerating nutrient remobilisation and leaf senescence (Dai et al., 1999), resulting in a shortened grain filling period contributing to grain yield losses (Howarth et al., 2008; Sanchez-Bragado et al., 2014, 2016).

While the early TCA cycle intermediates citrate, cis-aconitate, and isocitrate are gradually reduced over time, succinate, fumarate and malate accumulate which could be a result of catabolism of amino acids and other compounds, since their break down products may be fed into the TCA cycle through their conversion to the key C-skeleton, 2-oxoglutarate (Araújo et al., 2011). The gradual increase of these TCA intermediates is concomitant with the decrease in most other compounds, thus speculatively indicating both, degradation and export. Significant increases of citrate and fumarate levels could be detected in wheat phloem exudates extracted immediately below the head on the peduncle using aphid stylectomy (Palmer et al., 2014) showing that these TCA cycle intermediates are transported to the developing seed to provide carbon skeleton for in seed biosynthetic processes. As a result, their availability affects seed biochemistry and consequently yield.

Nitrate contents in the leaves decreased at 2 wpa and parallel to this the first decreases in chlorophyll contents announced the start of senescence and transport of solutes to the developing grain. This highlights the tight regulation of C/N ratio with the onset of senescence (Foyer et al., 2003; Raven et al., 2004; Nunes-Nesi et al., 2010; Mcallister et al., 2012; Bloom, 2015) as sucrose, glucose, fructose, and 1-kestose levels decrease with onset of senescence assumingly serving as precursors of storage carbohydrates or substrates for other anabolic pathways.

Throughout the post-anthesis time-course, the amino acid response patterns can be assigned to three groups. Glu was the major amino acid pool in leaves of all plants at anthesis which is in accordance with other studies (Lopes et al., 2006; Howarth et al., 2008). The first group of amino acids (Glu, Gln, Asp, Asn, Gly, Ser, Thr, and Ala) are high at anthesis and are gradually decreasing over time with the amplitude being directly related to N-supply. These decreases match those of sucrose, glucose, fructose and hence citrate, cis-aconitate, and isocitrate which can be interpreted additional to transport as consumption as precursors for amino acid biosynthesis or other biosynthetic pathways. Secondly, high levels of shikimate decreased strongly with development (as group 1) at the onset of anthesis, while the downstream aromatic acids (Trp, Tyr, Phe) increased and are apart from Tyr not even fully depleted from the senescent leaf tissue. Aromatic amino acid levels are very low at the beginning of anthesis. This inverse correlation of shikimate to aromatic amino acids (Guillet et al., 2000) and that aromatic amino acids only deplete upon long term N limitation (Schlüter et al., 2012) has been described previously. Trp, Tyr, and Phe displayed a strong N-dependent increase and a strong accumulation in leaves over the developmental period up to 4 wpa. The third group of amino acids (Leu, Val, Ile, Lys, His, and Arg) resemble the aromatic amino acid group as the contents peak around 3 wpa and then decrease with senescence. Again all of them are positively correlated to N supply. The concomitant decrease of nitrate at 2–3 wpa, even under N350 conditions, can be interpreted as consumption for amino acid biosynthesis.

Phosphate was one of the metabolites displaying the strongest increases in N0 treated plants (Figures 7, 9), and correlated negatively with straw and grain yield (Figure 8). Other nutrient ions do hardly respond to nitrogen supply levels or development. Nitrate itself only accumulates strongly under N350 fertilisation, while at all other conditions N is used for biosynthetic purposes and not stored in the vacuole. Ammonium contents are inconsistent in this study showing no obvious pattern. The high phosphate concentration in the leaves of N0 treated plants could be a result of continued phosphate uptake in plants which had a reduced growth. These results are in agreement with studies done in maize (Schlüter et al., 2012; Schluter et al., 2013), Medicago (Sieh et al., 2013), and Arabidopsis (Watanabe et al., 2013; Bielecka et al., 2014). Moreover, former studies could show that the N starvation-induced increase in phosphate levels and a readjustment of Pi homeostasis dominated the transcriptional changes recorded under N deprivation (Scheible et al., 2004; Morcuende et al., 2007; Bielecka et al., 2014). The increased phosphate contents in leaves of plants grown on low N can be explained as a result of the major impact nitrogen deficiency exerts on the plant's energy metabolism, i.e., the photosynthetic apparatus. A faster growth rate can only be achieved by increasing the plant N concentration. In this case the plant will first build the photosynthetic machinery before investing in the production of structural material to increase root growth. For P, (which largely remains in its oxidised form) a smaller portion is incorporated into the machinery of the plant's energy metabolism, so that, if the leaf lacks N, this leads to retarded growth, because carbohydrate backbones cannot be used for the biosynthesis of amino acids, proteins, and energy-rich compounds containing P such as ATP, leading to P accumulation. This means, residual N in soil (N0) depicts already an N-starvation condition, while at N100 and higher sufficient N is available to allow growth as seen also by the agronomical parameters (Figure 1, Supplemental Figure S1). P and N starvation share a substantial overlap of transcriptional responses (Watanabe et al., 2013) asking for a correlated regulation. Although not investigated here, N and S metabolism share a great deal of overlap (Hesse et al., 2004) and S starvation also results in phosphate accumulation (Watanabe et al., 2010) while N supply conditions here had no or little effect on sulphate tissue levels.

It has also been documented that the transport of sucrose from the shoot to the root stimulates phosphate transporters to further enhance phosphate uptake (Liu et al., 2005; Karthikeyan et al., 2007; Dasgupta et al., 2014). Thus, far, our understanding of phosphate over-accumulation and toxicity is limited to studies performed in *Hakea prostrata* (Kuppusamy et al., 2014; Prodhan et al., 2016), but the necessity for unravelling the underlying processes of nutrient cross-talk becomes more and more important in the face of future nutrient supply issues.

This investigation demonstrated that nitrogen starvation has wide-ranging effects on primary metabolism that are further influenced by processes occurring during post-anthesis development and defines grain yield. This study provides a metabolic map of those primary metabolites and nutrient ions that could be annotated in this study in response to N level during grain filling which will be useful for breeding for generating genetic diversity for improving nutrient use efficiency in general, and more specifically NUtE.

The major findings in this study can be summarised as follows:

This analysis could identify metabolites which are either responsive to nitrate availability, development or both. We could not identify genotypic markers for the agronomically identified traits high and low NUtE and early and late senescence probably due to the narrow genetic composition of the breeding lines or due to general processes of wheat metabolism thriving at keeping metabolome homeostasis.

The nitrogen fertiliser level was a strong determinant of the %N in the grain and straw. Plants grown on nitrogen poor soil delivered very poor yields, while N levels of 100 kg/ha doubled the yield increase in comparison to the unfertilised plants but with low pervcentage of N content in both, straw and grain. Grain yields of plants that received more than 200 kg/ha nitrogen did not increase any further. It was possible to identify metabolites positively correlating to yield increase or %N in the grain or straw. Amino acids were expectedly affected strongest by perturbations in N level and displayed a strong positive correlation to yield attributes.

## Author contributions

RH and MH: designed the research; EH, PB, MW, and AE: performed the research; EH, PB, MW, GD, AE, JK, and DW: analysed the data; EH and RH: wrote the paper.

### Conflict of interest statement

The authors declare that the research was conducted in the absence of any commercial or financial relationships that could be construed as a potential conflict of interest.

## Supplementary material

Supplementary Table S1 ANOVA (*p*-values) and FDR correction upon individual metabolites. ANOVA examined the interaction between the three different variables (genotype, treatment, and sampling time point) and their combinations on the metabolites measured in the experiment.
