# Supplementary figures and images for: Characterization of the Wheat Leaf Metabolome during Grain Filling and under Varied N-Supply
Source: Front Plant Sci. 2017 Nov 29;8:2048. doi: 10.3389/fpls.2017.02048 (PMC5712589; doi:10.3389/fpls.2017.02048)

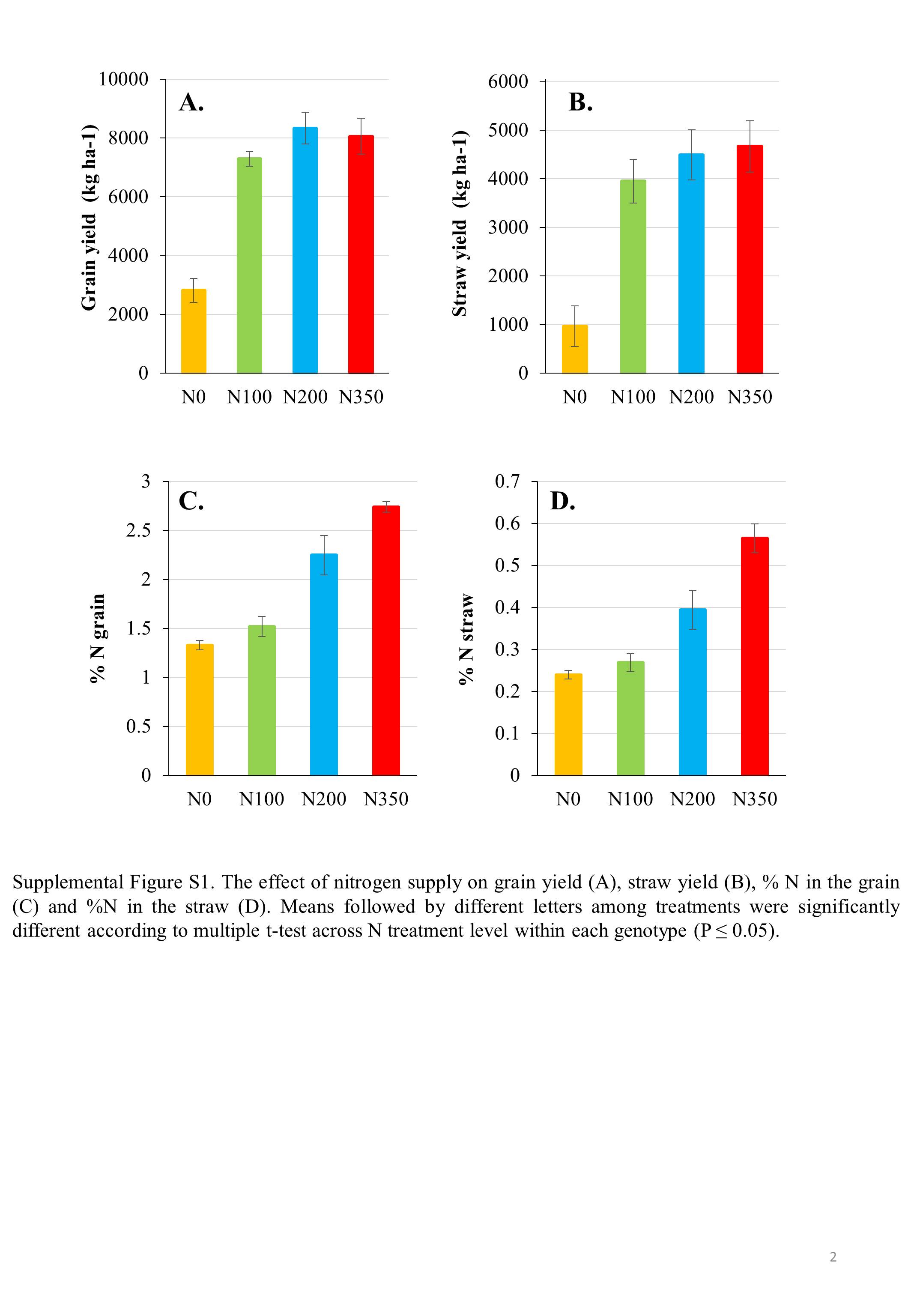

Supplement: Supplementary file 2 [file Image1.JPEG]

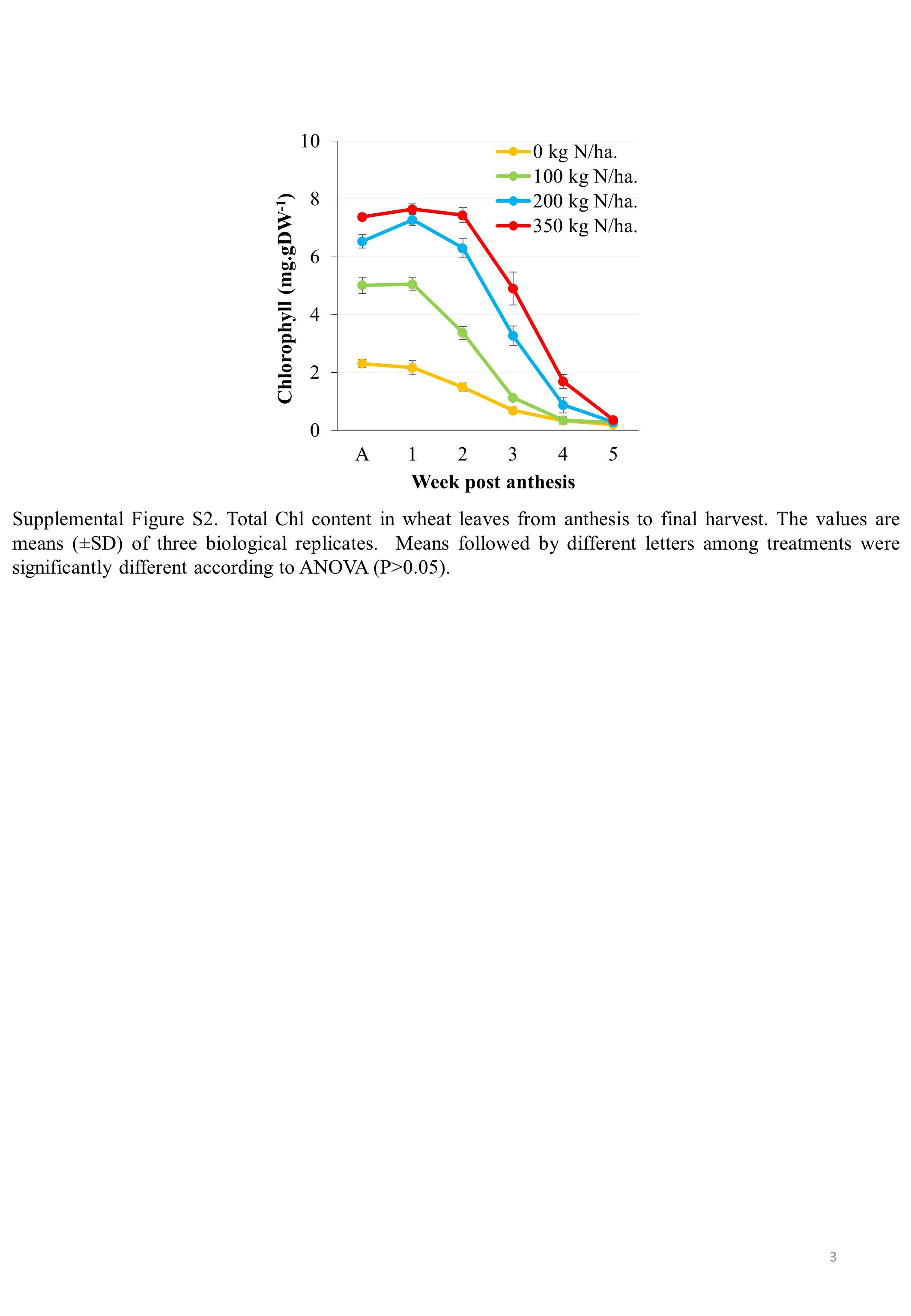

Supplement: Supplementary file 3 [file Image2.JPEG]
